# Comparison of Grafting Success Rate and Hearing Outcomes between Primary and Revision Tympanoplasties 

**Published:** 2019-01

**Authors:** Mohammad Faramarzi, Mahmood Shishegar, Saeed Reza Tofighi, Hadi Sharouny, Raman Rajagopalan

**Affiliations:** 1 *Otorhinolaryngology Research Center, Shiraz University of Medical Sciences, Shiraz, Iran.*; 2 *Otorhinolaryngology Research Center, Tehran University of Medical Sciences, Tehran, Iran.*; 3 *Department of Otorhinolaryngology Head and Neck Surgery, University Malaya Medical Center, University of Malaya, Kuala Lumpur, Malaysia.*

**Keywords:** Hearing, Myringoplasty, Tympanoplasty, Treatment outcome, Tympanic membrane perforation

## Abstract

**Introduction:**

There are a few studies that compare the outcomes between primary and revision tympanoplasties. The purpose of the present study was to compare the results of type I tympanoplasty (i.e., synonymous to myringoplasty) and revision myringoplasty based on the closure of tympanic membrane perforation and hearing improvement.

**Materials and Methods:**

This prospective single-blind study was carried out on a total of 240 patients with tympanic membrane perforation at a tertiary referral center. The subjects underwent primary or revision myringoplasty. Grafting success rate and hearing results were measured and the comparison between the primary and revision groups was drawn.

**Results:**

Grafting success rate was reported as 96.6% (112 out of 116 cases) for myringoplasty, while in revision myringoplasty the success rate of 78.2% (97 out of 124 patients) was achieved (P=0.001). Speech reception threshold was 23.1±9.2 dB and 24.9±13.1 dB in the primary and revision groups, respectively (P>0.05). However, the percentage of air-bone gap on audiometry≤20 dB were 83.8% and 76% in the primary and revision groups, respectively (P=0.26).

**Conclusion:**

The findings of the present study have shown that although grafting success was reported significantly better in myringoplasty (tympanoplasty type 1), compared to that in revision myringoplasty, it did not reveal any superiority over revision tympanoplasty regarding the hearing outcomes. No consensus was achieved due to a great number of controversies in the literature.

## Introduction

The prevalence of chronic otitis media (COM) is still high worldwide, especially in the developing countries (1,2). Cholesteatoma and granulation are prevalent potential risk factors in complicated COM (3). The COM can be divided in two major groups. The first group is the active type, including subtypes with and without granulation tissue or cholesteatomas. The other group is inactive type consists of subtypes with dry tympanic membrane (TM) perforation, retraction pocket, adhesive TM, and the ossicular resorption or fixation. The majority of otologists acknowledged that the success rate of surgery could considerably be affected by these types and subtypes (4). 

In a retrospective study, it was shown that the prevalence rate of tympanoplasty was reported as 2.97 per 100,000 patients in 1996. Then, it sharply increased to 26.70 in 2001 and slowly decreased to 16.61 in 2007. On the other hand, revision tympanoplasty had a constant rate of 0.37 per 100,000 patients (5). According to the literature, it was revealed that several factors can affect the surgery, such as the size and site of TM perforation, presence of otorrhea, cholesteatoma, granulation tissue, tympano- sclerotic plaque, tympanoplasty with or without mastoidectomy, canal wall-down versus canal wall-up mastoidectomy, age of patients, ossicular status, condition of the contralateral ear, smoking, surgeon’s experience, and revision surgery (6,7). 

Based on the results of a study, it was demonstrated that cholesteatoma is not a significant prognostic factor in grafting success rate (8). Mainly some prospective studies have addressed the issue of revision surgery regarding the COM as a prognostic factor for grafting success rate. Accordding to the literature it was reported that revision surgery has a less successful outcome, compared with primary surgery (9-13); however, not all the studies support the aforementioned idea (14). The abovementioned controversies necessarily imply the lack of consensus on the prognostic factors of tympanoplasty. Despite the finding of the previous studies, whether the anatomic and functional outcomes of primary surgery are better than those of revision surgery is still not clear. Therefore, the present study aimed to compare the short-term outcomes of primary and revision surgery by the elimination of other prognostic factors.

## Materials and Methods

A prospective single-blind study was carried out on the patients that underwent primary or revision tympanoplasty during January 2012 to October 2016 at Dastgheib University Hospital in Shiraz, Iran. The study protocol was approved by the university ethics committee (registration no.: ec-p-92-6142). All procedures were carried out by the first author (M.F.). All the patients’ ears were dry for at least three months prior to the operation. The post-auricular approach and temporalis fascia graft with underlay technique were utilized in all the operations. 

The inclusion criteria were all primary and revision tympanoplasties in adults with the size of perforation≥50% of tympanic membrane area with normal ossicular chain. The duration between the primary and revision surgery was at least 6 months. The exclusion criteria were subjects under 14 years old, smokers, the cases with contralateral ear disease, cholestatoma, tympanosclelerotic plaque or granulation tissue in middle ear, simultaneous mastoid surgery, and preoperative medical problems, such as asthma, diabetes, cardiovascular disease, and chronic liver or renal diseases.Grafting success rate was defined as dry ear with intact TM in a well-aerated mesotympa- num and the absence of retraction in tympanic membrane. Post-operative follow-up ranges between 6-10 months. The postoperative microscopic otoscopy of the ears was performed by another otolaryngologist that was blind to the nature of operation. The postoperative findings were recorded in prepared forms. Air conduction, bone conduction, and air-bone gap (ABG) on audiometry at the frequencies of 0.5, 1, 2 and 3 kHz, and the speech reception threshold (SRT) were measured before and after the surgery. 

Data analysis was performed by SPSS software (version 18.0). For continuous variables, independent groups were compared using independent t-test or Mann-Whitney U test, whereas Pairwise comparison was drawn using paired t-test or Wilcoxon signed-rank test. The relationships between categorical variables were assessed by chi-square test or Fisher’s exact test. The main criterion for statistical significance was considered at P<0.05 for all hypothesis testing.

## Results

A total of 125 patients in the myringoplasty group and 140 subjects in the revision myringoplasty group were included in this study. Out of all cases, 9 patients in the primary group and 16 participants in revision group did not attend follow-ups.

Eventually, 116 subjects enrolled in the primary tympanoplasty group and 124 in the revision tympanoplasty group. 

The mean ages of the revision group and primary group were 35.9±12.1 (age range: 18-58) and 37.2±12.7 years (age range: 14-62), respectively (P=0.79). There were 76 (61.3%) female and 48 (38.7%) male cases in revision group, while the number of female and male cases were reported as 78 (67.2%) and 38 (32.8%) in primary group (P=0.33). In revision group, 59 (47.6%) and 65 (52.4%) cases underwent right and left ear operations, respectively, while in primary group, 56 (48.3%) and 60 (51.7%) experienced right and left ears operations, respectively (P=0.56). Both revision and primary groups were similar in terms of the age, gender, ear operations, and size of the perforation and there were statistically no significant differences between the revision and primary groups in the baseline characteristics (Table 1).

**Table 1:** Subject demographics in the study groups

	**Revision myringoplasty** **(n=124)**	**Primary myringoplasty** **(n=116)**	**P Value**
Age in years, mean (SD)	35.9±12.1	37.2±12.7	P>0.05
Gender, no. (%)			
Male	48 (38.7%)	38 (32.8%)	P>0.05
Female	76 (61.3%)	78 (67.2%)	P>0.05
Operated ear, no. (%)			
Right	59 (47.6%)	56 (48.3%)	P>0.05
Left	65 (52.4%)	60 (51.7%)	P>0.05

According to the results, it was revealed that grafting success rates regarding the complete closure of the perforation were reported as 78.2% (97 out of 124 patients), and 96.6% (112 out of 116 patients) for revision myringoplasty and primary myringoplasty, respectively. In this regard, there were statistically significant differences between the two groups (P=0.001). 

There were 50 and 68 cases with normal ossicular chain in revision and primary groups, respectively. There were no significant differences between the two groups regarding the preoperative audiometry parameters (Table.2). The post-operative SRT in the primary group was 23.1±9.2 dB, while it was 24.9±13.1 dB in the revision group; however, the difference was not statistically significant (P=0.38). Moreover, the SRT mean gains were reported as 14±9.3 and 16.2±8.2 dB in revision and primary groups, respectively. In addition, the difference was not statistically significant (P=0.24). Although the postoperative ABG of the primary group at 500-3000 and 4000 Hz were lower than that in the revision group. There was no statistically significant difference between the two groups in postoperative ABG at all frequencies (Table.2). 

**Table 2:** Comparison between the pre and post-operative hearing outcomes between two study groups

	**Revision Group(n=50)**	**Primary Group (n=68)**	**P Value**
Pre-operative			
SRT (dB)	38.9±11.7	39.3±11.3	0.52
ABG at 500-3000 (dB)	29±9.8	29±9.5	0.98
ABG at 4000 (dB)	27.8±11.2	28.4±10.9	0.75
Post-operative			
SRT (dB)	24.9±13.1	23.1±9.2	0.38
ABG at 500-3000 (dB)	15.8±8.1	15.3±6.6	0.84
ABG at 4000 (dB)	17±11.5	16.3±10.2	0.73
SRT gain (dB)	-14±9.3	-16.2±8.2	0.24
ABG difference at 500-3000 (dB)	-13.1±9.4	-13.6±9.2	0.77
ABG difference at 4000 (dB)	-10.8±5.7	-12.1±6.3	0.62

SRT: Speech Reception Threshold; ABG: Air-Bone Gap

**Fig 1:**
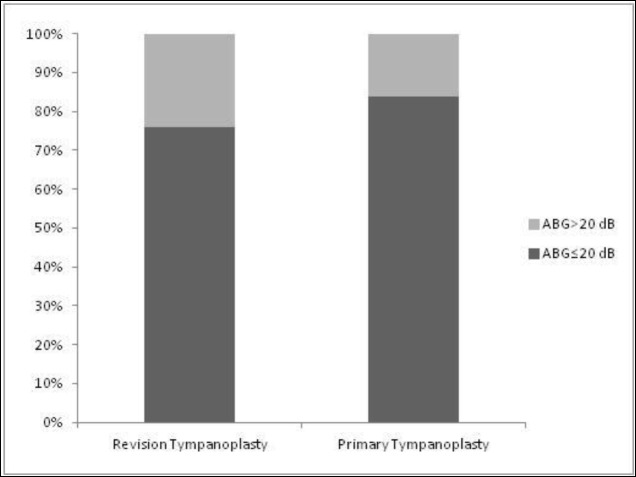
Post - operative Air-Bone Gap in two study groups.ABG: Air-Bone Gap

Another statistical analysis revealed that the postoperative ABG of less than 30 dB was achieved in 46 (90%) and 64 cases (94.1%) in revision and primary groups, respectively. In addition, there were 38 cases (76%) with a postoperative ABG≤20 dB in the revision group; however, there were 57 cases (83.8%) with a postoperative ABG≤20 dB in the primary group. The primary group gained a better hearing result in the ABG parameters of audiometry, compared to that in the revision group; however, the difference was not statistically significant (P=0.26) (Fig. 1).

## Discussion

According to the literature, it was revealed that there is a wide range of grafting success rate (5%-97%) with temporalis fascia in myringoplasty (7,15-18). Nevertheless, the majority of studies have reported the grafting success rate of about 85%, while other studies have announced approximately 75% (6,19-29). A study conducted on 389 revision tympanoplasty cases showed the grafting success rate of 90% (30). In another study, a grafting success rate of 95% was reported using fascia graft; however, there were only 19 revision cases in the study (30). In a study performed in Finland, grafting success rate in revision myringoplasty was 79%, compared to 88% in primary surgery, which did not significantly differ (11).

 In another study, the anatomic and hearing results were compared in two groups of patients with chronic suppurative otitis media versus dry tympanic membrane perforations that underwent tympanoplasty without mastoidectomy. The results of the mentioned study revealed that revision surgery has a negative effect on grafting success rate (81.6% in the revision group versus 92.8% in the primary group) (31). In another study carried out on grafting success rate in various tympanoplasty operations in patients with chronic non-cholesteatomatous otitis media, the grafting success rate was reported as 93.6% and 90.2% regarding the subjects in the primary and revision tympanoplasty groups, respectively. There was no evidence regarding the increased risk of failure in revision tympanoplasty cases, compared to primary tympanoplasty patients (32). The results of majority of studies revealed that revision surgery has a negative effect on grafting success rate (9-13); however, a limited number of studies announced there was no significant difference in grafting success rate between the primary and revision tympanolasties (11,14, 32).In the present study, the grafting success rates were 78.2% and 96.6% in the revision and primary tympanoplasty surgeries, respectively (P<0.05). Probably, it may be related to the compromised vascularity of the middle ear in the revision surgery. On the other hand, the hearing results in terms of the postoperative SRT and ABG closure were not statistically significant. There are controversies regarding the choice of graft material in revision surgery, although cartilage tympanoplasty was used in some studies as the material of choice for revision operations or large TM perforations (33-41). The results of a study showed grafting success rate of 91% in 35 revision cases using the scar tissue as a grafting material (33). Another study indicated an 86% grafting success rate in revision tympanoplasty using temporalis fascia, tragal perichondrium, or periosteum, which is slightly better than the grafting success rate in the present study (42). 

In a retrospective study conducted on 114 patients undergoing myringoplasty and 11 cases experiencing revision myringoplasty, the exclusion criteria were mastoidectomies, cholesteatoma, and ossicular reconstruction. Cartilage was the most commonly used graft material (in 82.5% of the cases). An overall grafting success rate of 71.1% was obtained during 6 months follow-up. However, higher grafting success rates was achieved in primary surgeries, compared to those in revision operations (72% versus 54%); however, due to a small sample size in revision surgeries, no statistically significant differences could be discovered between the two types of primary or revision tympanoplasty regarding postoperative grafting success rate and hearing results (17).

The results of a study performed in Turkey indicated tympanoplasty on patients with tympanic perforations with a size larger than 50% of tympanic membrane that was reported with a success rate of about 84%. However, the smaller perforations were noticed with the success rate of about 92% and the difference was statistically significant (29). In another case series, the grafting success rate was reported about 65% in cases with tympanic perforations with the size of >50%, while this rate was 82% in smaller perforations (9). 

Based on the evidences, it was stated that the size of perforation is not a risk factor (43). On the other hand, other studies reported that the size of perforation affects the grafting success rate (9,29,44). In the present study, only the patients with their tympanic perforations≥50% of tympanic membrane area were included. Although patient selection with larger TM perforation may cause higher graft failure and make it possible to draw comparison between the two groups. 

In the present study, a successful hearing result of the ABG within 20 dB was achieved as 76% and 83.8% in revision and primary tympanoplasties, respectively. In addition, the results of this study showed that the postoperative ABG of less than 30 dB in 90% and 94.1% of revision and the primary tympanoplasties, respectively (P>0.05). According to the results of a study it was revealed that a residual ABG of≤30 dB was obtained in 70.3% (n=41) of the cases only after revision tympanoplasty (30). 

In a retrospective study, no significant differences were identified regarding the hearing results in patients undergoing revision tympanoplasty with cartilage-perichondrium versus perichondrium (32). In a study conducted with different graft materials, the ABG within 20 dB was achieved in 69.5% and 81.1% of the cases in revision and primary tympanoplasties, respectively (33). One of the probable causes of difference in the ABG between primary and revision tympanoplasty cases in the mentioned study was due to using scar tissue as a graft material in revision operations, while temporalis fascia is utilized in primary surgeries. 

Using various materials as graft for revision tympanoplasty and not paying particular attention to perform studies, including several confounding variables, such as tympanic membrane perforation size, tympanosclelerotic plaques, and ossicular continuity might cause various results in grafting success rate and hearing outcome.

The main limitation of the present study was the relatively short duration of the follow-up. Another limitation of this study was that only the tympanic perforations with a size of>50% were evaluated. The use of the temporalis fascia graft for primary and revision tympanoplasties would result in better effects regarding the hearing outcome between the two groups; however, it may cause lower grafting success rate in revision tympanoplasty, compared to that in primary tympanoplasty.

## Conclusion

 The findings of the present study have shown that although grafting success is significantly higher in primary myringoplasty (type 1 tympanoplasty), compared to that in revision myringoplasty; however, it did not reveal any superiority in revision tympanoplasty regarding the hearing outcomes. No consensus emerged due to major controversies in the related literature. Further studies with a larger population, different graft materials, various sizes of perforation and longer follow-ups, are needed to precisely evaluate and compare the efficacy and safety related to these procedures.
